# Supplementary material for: Effects of Capsaicin on Glucose Uptake and Consumption in Hepatocytes
Source: Molecules. 2023 Jul 6;28(13):5258. doi: 10.3390/molecules28135258 (PMC10343879; doi:10.3390/molecules28135258)
Supplement: Supplementary file 1 [file molecules-28-05258-s001.zip › molecules-2468840-supplementary.pdf]

Table S1. Statistical results of genome mapping and quantity of reads in transcriptome sequencing.

	RNA-Seq	Clean Reads	Mapped Reads	Map Ratio
4 h	DMSO-1	9,375,266	8,650,557	71.86%
	DMSO-2	9,316,776	8,156,837	65.79%
	CAP-1	9,794,097	9,027,219	65.05%
	CAP-2	10,079,013	8,853,405	64.05%
12 h	DMSO-1	10,325,697	9,016,398	64.96%
	DMSO-2	9,127,116	8,388,732	65.03%
	CAP-1	8,756,819	7,677,978	65.93%
	CAP-2	12,065,171	10,949,142	67.43%
24 h	DMSO-1	12,656,710	11,501,152	66.33%
	DMSO-2	13,460,104	12,248,694	65.79%
	CAP-1	12,030,339	10,873,020	68.27%
	CAP-2	11,600,746	10,538,117	66.93%

Table S2. The specific primers of the genes used for quantitative PCR.

Gene	Primer Sequences	
ACSS2	F: GAACATGAGGCTGTTGCAGA	R: ATGGGGCCAATCTTTTCTCT
ASNA	F: TGTCTCTGCCACCAGAAATG	R: GCCATCATTGCATCATCAAC
CTH	F: TGAATGGCCACAGTGATGTT	R: CCATTCCGTTTTTGAAATGC
FOS	F: AGAATCCGAAGGGAAAGGAA	R: CTTCTCCTTCAGCAGGTTGG
IGFBP3	F: CCTGCCGTAGAGAAATGGAA	R: AGGCTGCCCATACTTATCCA
IL-6	F: AGGAGACTTGCCTGGTGAAA	R: CAGGGGTGGTTATTGCATCT
NXF1	F: GATGTGGCAATGAGTGATGC	R: TTCTTTGAGGTCCCATCCTG
PCK2	F: TGTGGGGGATGATATTGCTT	R: TGGTCTCAGCCACATTGGTA
PHGDH	F: GGCTCAATGGAGCTGTCTTC	R: TTCAGTCACATGCTGCTTCC
PSAT1	F: AGCAGGAAGGTGTGCTGACT	R: GTAGGAGGCATCTGGGTTGA
SLFN5	F: CCGACAAAATCCTCCACCTA	R: CTTTGGTGAAAAGCACAGCA
VEGF1	F: AAGGAGGAGGGCAGAATCAT	R: ATCTGCATGGTGATGTTGGA
β-actin	F: CCTTCCTGGGCATGGAGTC	R: TGATCTTCATTGTGCTGGGTG
